# Knockdown of lncRNA XIST Ameliorates IL-1*β*-Induced Apoptosis of HUVECs and Change of Tissue Factor Level via miR-103a-3p/HMGB1 Axis in Deep Venous Thrombosis by Regulating the ROS/NF-*κ*B Signaling Pathway

**DOI:** 10.1155/2022/6256384

**Published:** 2022-11-18

**Authors:** Guangxin Cao, Hua Zhou, Dong Wang, Lei Xu

**Affiliations:** ^1^Department of Vascular Surgery, Weifang People's Hospital, Weifang City, Shandong, China 261000; ^2^Department of Vascular Surgery, Shandong Provincial Hospital Affiliated to Shandong First Medical University, No 324, Jingwuweiqi Road, Jinan City, Shandong, China 250021

## Abstract

**Background:**

The effect of lncRNA X inactive-specific transcript (XIST) inducing cardiovascular diseases on deep vein thrombosis (DVT) and its mechanism has not been reported. In this study, we uncovered the mystery that lncRNA XIST causes DVT with HUVEC dysfunction.

**Method:**

The expression levels of lncRNA XIST and miR-103a-3p were detected by qRT-PCR, and HMGB1 expression was determined by qRT-PCR and western blot. The correlations among the expression levels of lncRNA XIST, miR-103a-3p, and HMGB1 were determined by Spearman's rank-order correlation test. XIST siRNA (si-XIST) was transfected into HUVECs to knock down the intrinsic expression of lncRNA XIST. The influences of si-XIST on interleukin-1 beta- (IL-1*β-*) treated HUVEC viability and apoptosis and the level of tissue factor (TF) were detected by MTT, flow cytometry, and ELISA kit, respectively. The relationships between lncRNA XIST, miR-103a-3p, and HMGB1 were predicted by the Encyclopedia of RNA Interactomes (ENCORI) database and verified by dual luciferase reporter assay. The effects of lncRNA XIST and miR-103a-3p on HMGB1 expression were detected by qRT-PCR, western blot, and immunofluorescence analysis. The levels of ROS/NF-*κ*B pathway-related proteins were detected to study the regulatory mechanism of lncRNA XIST/miR-103a-3p/HMGB1 on IL-1*β*-treated HUVECs apoptosis and change of TF level.

**Results:**

The upregulated expression levels of lncRNA XIST and HMGB1 and downregulated level of miR-103a-3p were found in the plasma of DVT patients and IL-1*β*-treated HUVECs. Si-XIST promoted cell viability and inhibited HUVEC apoptosis and ameliorated the change of TF level triggered by IL-1*β*. lncRNA XIST sponged miR-103a-3p and miR-103a-3p targeted HMGB1. Si-XIST inhibited the ROS/NF-*κ*B pathway to suppress HUVEC apoptosis and ameliorate the change of TF level induced by IL-1*β* via the miR-103a-3p/HMGB1 axis.

**Conclusion:**

lncRNA XIST sponged miR-103a-3p improving HMGB1 expression to exacerbate DVT by activating the ROS/NF-*κ*B signaling pathway. Our findings indicated that lncRNA XIST can be used as a potential therapeutic target in DVT.

## 1. Introduction

Deep vein thrombosis (DVT) is one of the serious cardiovascular diseases (CVDs) [1], which is abnormal blood coagulation in the deep vein. 10 million DVT cases are found in China every year [2]. Only a few DVT patients will recover by themselves based on the spontaneous dissolution of the thrombus, and most DVT patients need operations to improve their life qualities. Endovascular or open surgery was conducted, but these therapeutic methods have side effects and cannot be applied to all patients [3]. Therefore, the development of new therapies is necessary.

Some researchers reported that vascular wall injury and blood flow change (abnormal blood coagulation) are the main factors leading to DVT [2]. The vascular wall injury is caused by the injury of the vascular endothelial cells. Human umbilical vein endothelial cells (HUVECs) have been used in plenty of studies on anticoagulation function. The injury and apoptosis of HUVECs make cells lose the normal anticoagulation function. Meanwhile, it also promotes the formation of thrombus [2]. Moreover, coagulation disorders are associated with blood clot formation to exacerbate thrombus [4]. Long noncoding RNAs (lncRNAs) are longer than 200 nucleotides without protein-coding capacity [5]. The competitive combination of lncRNAs and microRNA (miRNA) regulates the expression of messenger RNA (mRNA) [6], which further regulates many biological processes [7, 8]. Some lncRNAs related to CVDs might be involved in DVT development. Hyperglycemia and hypertension can injure the vascular endothelial cells to form thrombus due to metabolic disturbance. Atherosclerosis results in the narrowing of the vascular lumen and the increase in blood viscosity, which may lead to thrombus formation. The expression level of lncRNA XIST was higher in pulmonary hypertension compared with the healthy person [9]. Meanwhile, lncRNA XIST was overexpressed to enhance gestational diabetes mellitus by sponging miR-497-5p/forkhead box protein 1 (FOXO1) [10]. What is more, the knockdown of lncRNA XIST could reduce the vascular wall injury induced by low-density lipoprotein via miR-204-5p/toll-like receptor 4 (TLR4), which attenuated atherosclerosis [11]. Therefore, XIST lncRNA expression might be significantly increased in DVT.

miRNAs are short (18-22 nt) RNAs without coding ability. miRNAs can bind to the 3′ untranslated regions (UTR) in mRNA to induce gene silence, which inhibits the translation or promotes mRNA degradation. It was found that miRNAs participated in the process of many diseases including DVT by regulating the expression of genes that are crucial for the pathogenesis of the disorders [12]. There was a low expression of miR-103a-3p reported in patients with thrombosis [13]. In addition, it has been reported that low expression level of lmiR-103a-3p blocked normal functions of endothelial progenitor cells **(**EPCs, the source of endothelial cells) under the regulation of phosphatase and tensin homolog **(**PTEN) in DVT [12]. The development of lower-extremity deep venous thrombosis (LEDVT) was reduced by inhibiting chemokine 12 (CXCL12) with the increase of miR-103a-3p expression [14].

High-mobility group box 1 (HMGB1) was released with the activation of innate immunity. HMGB1 is not only the main regulator of the coagulation cascade but also participates in the apoptosis of cells [15–18]. The expression level of HMGB1 was enhanced significantly in DVT [15]. HMGB1 was proven to induce apoptosis and inflammatory reaction. Meanwhile, the risk of DVT was increased in patients diagnosed with coagulation disorders followed by the injury of HUVECs undergoing apoptosis [19–21]. The reactive oxygen species (ROS) signal pathway was activated based on the abundant HMGB1. In addition, the inflammatory factor interleukin-1 beta (IL-1*β*) also enhanced the ROS level by activating the inflammatory cascade [22–24]. ROS made a great contribution to coagulation impairment by elevating the level of TF, and abnormal blood coagulation increased the risk of DVT [1, 25]. ROS also stimulated the apoptosis of HUVECs to promote DVT via the NF-*κ*B signaling pathway [2, 26].

The direct binding relationship between lncRNA XIST and miR-103a-3p was predicted by the Encyclopedia of RNA Interactomes (ENCORI) miRNA database (https://starbase.sysu.edu.cn/agoClipRNA.php?source=mRNA). miR-103a-3p targeting HMGB1 reduced sepsis-induced liver injury [27]. Considering the above information, we will explore the connection between lncRNA XIST and miR-103a-3p/HMGB1 axis in IL-1*β*-treated HUVECs and the involvement of the underlying mechanisms.

## 2. Material and Methods

### 2.1. Clinical Samples

The plasma samples from 20 DVT patients and 20 healthy volunteers were collected from the Shandong Provincial Hospital Affiliated to Shandong First Medical University. The included DVT patients were confirmed by color doppler ultrasound and lower extremity angiography without a history of hypertension, diabetes mellitus and other chronic diseases. The informed consent was signed by all patients and healthy persons. Blood samples were drawn in 5 mL tubes containing sodium citrate and were shipped to laboratories. To obtain the plasma, the blood samples were immediately centrifuged at 3000 rpm for 10 min and then frozen at -70°C until further analysis. Meanwhile, our study met the ethical requirements and obtained approval.

### 2.2. Cell Culture

The HUVECs were acquired from American Type Culture Collection (ATCC, USA) and cultured in the endothelial cell medium with the addition of 5% FBS, 1% penicillin, and 10% endothelial cell growth supplement (Sigma, USA). The cultures were maintained in humidified atmosphere containing 5% CO_2_ at 37°C. HUVEC cultures at 80% confluency were used for further tests.

The HUVECs were incubated with no addition (control group) or different concentrations of IL-1*β* (2, 5, 10, and 20 ng mL^−1^) for different periods (24, 48, and 72 h).

### 2.3. MTT Assay

200 *μ*L of HUVECs were transferred into 96-well plates (3 × 10^3^ cells well^−1^). After incubation for 4 h with 10 *μ*L MTT (5 mg mL^−1^), the supernatant was discarded. The cells were treated with 100 *μ*L dimethyl sulfoxide. Finally, the absorbance was measured by a microplate reader at 490 nm.

### 2.4. Cell Transfection

siRNA against XIST (si-XIST) and si-NC was obtained from Gene Copoecia (Guangzhou, China) [28]. miR-130a-3p inhibitor, negative control of the inhibitor (inhibitor-NC), miR-130a-3p mimics, mimics NC, HMGB1 siRNA (si-HMGB1), and siRNA negative control (si-NC) were obtained from Gene Pharma (Shanghai, China) [12, 29]. The scrambled RNA was used as the control. The nontrasfected HUVECs served as the blank group. All of their sequences were listed in Table 1. The HUVECs were plated into 6-well plates. After 24 hours, the cultures were transfected using Lipofectamine 3000 reagent (Invitrogen, USA). After transfection, the cells were cultured in the nutrient-rich medium for 5 h. For the need of the planned experiments, cells were subcultured and maintained in a standard medium.

### 2.5. qRT-PCR

The total RNAs were extracted from HUVECs by TRIzol (Life Technologies, USA) reagent. Then, the RNAs were used to synthesize cDNA using the PrimeScript RT reagent Kit (Takara, Japan). The qRT-PCR was conducted with SYBR-Green qPCR Master Mix (Takara, Japan). The housekeeping reference gene was GAPDH, and the expression of relative RNA was determined by the 2^−△△Ct^ method. The primer sequences were listed in Table 2.

### 2.6. Western Blot

The proteins were attained by RIPA lysis buffer (Beyotime, China). Then, the proteins were separated by SDS-PAGE. After transferring onto the PVDF membrane, the blots were incubated in 5% nonfat milk in TBS. Next, the membrane was incubated at 4°C overnight with the primary monoclonal rabbit anti-human antibodies (HMGB1, 1 : 10000; Bax, 1 : 1000; Cleaved caspase 3, 1 : 500; Bcl-2, 1 : 1000; p65, 1 : 1000; p-p65, 1 : 1000; GAPDH, 1 : 10000; *β*-actin, 1 : 200) (Abcam, USA). After washing with TBST, the membrane was treated using the secondary polyclonal horseradish peroxidase- (HRP-) linked goat anti-rabbit IgG antibodies (1 : 1000) (Cell Signaling Technology, USA). Finally, the membrane was treated with an enhanced chemiluminescence reagent (Beyotime, China). The ImageJ software v1.48 was used for the quantification of protein bands.

### 2.7. Detection of Cell Apoptosis by Flow Cytometry

The apoptosis of HUVECs was determined using Annexin V-FITC/PI double staining kit (Beyotime, China). The sample was further analyzed using the FACSCanto flow cytometer (BD Biosciences, USA) [30].

### 2.8. TF Elisa

The HUVECs (2 × 10^5^) were placed into 6-well plates and lysed with cell lysis buffer containing 1% protease inhibitor cocktail for 5 h. After centrifuging (10000 g) for 10 min, TF level was detected by the Tissue Factor Quantikine ELISA kit (Bio-Techne Ltd, UK) [31].

### 2.9. Dual-Luciferase Reporter Assay

The direct binding ability of lncRNA XIST and miR-103a-3p was detected by Promega Luciferase Assay System (Promega, USA). The wildtype (WT) containing miR-103a-3p binding site and mutant (MT) without miR-103a-3p binding site of lncRNA XIST was subcloned into PsiCHECK2 luciferase reporter vectors, individually. Then, the PsiCHECK2 vectors and miR-103a-3p mimic or mimic-NC were cotransfected into the HUVECs. After 48 h, the relative fluorescence value (Luciferase/Renilla) was detected. A similar operation method was conducted to detect the direct binding ability of miR-103a-3p and HMGB1.

### 2.10. Immunofluorescent Analysis

The HUVECs were fixed in 4% paraformaldehyde then rehydrated. The cells were immersed in 0.2% triton in PBS for 5 min. Then, 2% bovine serum albumin was used to prevent the nonspecific binding of antibodies. After washing with PBS, the cells were incubated overnight with the primary monoclonal rabbit anti-human antibody (HMGB1, 1 : 250, Abcam, USA). Then, the secondary polyclonal goat anti-rabbit IgG antibody conjugated to Alexa Fluor 594 was applied (1 : 200, Abcam, USA). Meanwhile, the nuclei were stained with DAPI (Beyotime, China) for 2 minutes. The images of the cells were captured using a fluorescence microscope and analyzed using ImageJ software v1.48 [29, 32].

### 2.11. Reactive Oxygen Species (ROS) Analysis

The ROS level was examined by DCFH-DA staining (Sigma-Aldrich, USA). In brief, HUVECs (10^4^ cells) were plated on 96-well black plates and cultured overnight. Next, HUVECs were treated with 50 *μ*M DCFH-DA staining kit (Sigma-Aldrich, USA) for 20 minutes at 37°C. After washing with PBS, the fluorescence intensity indicating the ROS level was determined with a microplate reader [33, 34].

### 2.12. Statistical Analysis

Each experimental group of cells was cultured in triplicates, and the results were presented as mean ± standard deviation. Statistical analysis was performed using Student's *t*-test (between two groups) and one-way ANOVA (among multiple groups). Spearman's rank-order correlation test was used to calculate the correlation of lncRNA XIST and miR-103a-3p, miR-103a-3p, and HMGB1. *P* < 0.05 was considered statistically significant. The graphs were prepared using GraphPad Prism 6.0 software.

## 3. Results

### 3.1. Knockdown of lncRNA XIST Attenuated the Apoptosis of HUVECs and Resulted in a Decrease of IL-1*β*-Induced TF Level *In Vivo*

The baseline characteristics of DVT patients and healthy volunteers were in Table 3. At first, qRT-PCR was executed to detect the expression levels of lncRNA XIST, miR-103a-3p, and HMGB1 mRNA in the plasma of 20 DVT patients (DVT group) and 20 healthy persons (normal group).

**Figure 1:**
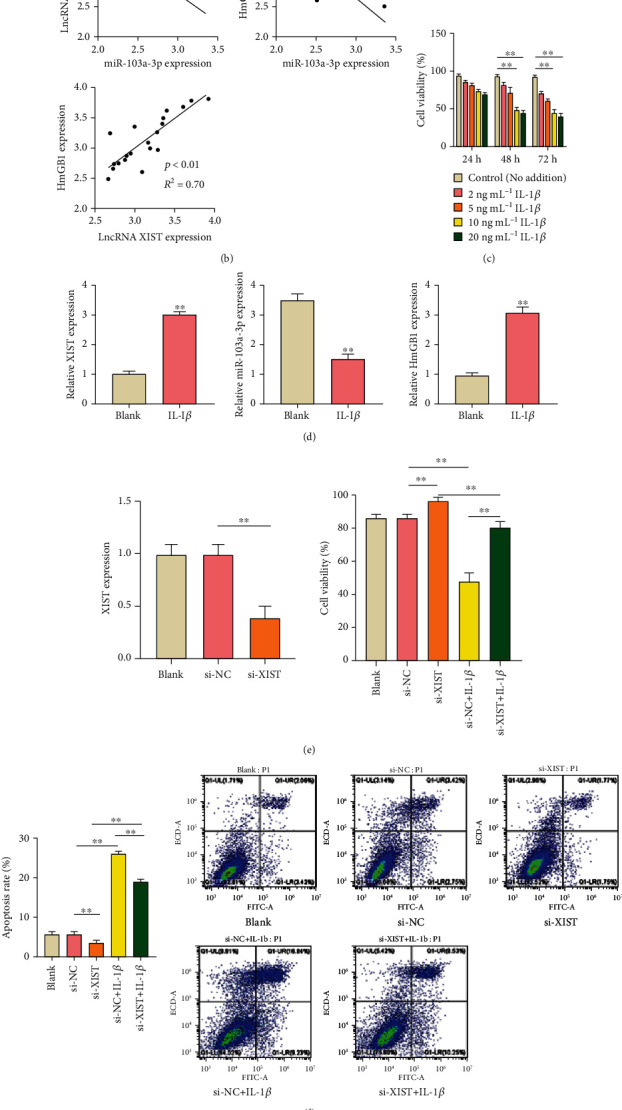
Knockdown of lncRNA XIST attenuated the apoptosis of HUVECs and resulted in a decrease of IL-1*β*-induced TF level *in vivo*. (a) The expression levels of lncRNA XIST and miR-103a-3p were detected by qRT-PCR, and the expression level of HMGB1 was analyzed by qRT-PCR and western blot. (b) The correlations among the expression levels of lncRNA XIST, miR-103a-3p, and HMGB1 were calculated by Spearman's rank-order correlation test. (c) The cell viability of HUVECs under IL-1*β* addition was determined by MTT assay. (d) The expression levels of lncRNA XIST, miR-103a-3p, and HMGB1 were measured under IL-1*β* treatment by qRT-PCR. (e) The expression level of lncRNA XIST in transfected HUVECs was analyzed by qRT-PCR, and the cell viability in transfected cells was detected by MTT assay. (f) The cell apoptosis was analyzed by Annexin V-FITC/PI double staining kit. (g) The expression levels of proteins related to apoptosis were detected by western blot. (h) The concentration of TF antigen was determined by the ELISA kit. The nontransfected HUVECs served as the blank group. ^∗^*P* < 0.05 and ^∗∗^*P* < 0.01.

The expression levels of lncRNA XIST and HMGB1 mRNA in DVT patients were higher than that in healthy persons. However, miR-103a-3p had a lower expression in DVT patients compared with healthy persons (*P* < 0.01) (Figure 1(a)). Meanwhile, the high expression level of HMGB1 protein (*P* < 0.01) in DVT patients was shown by western blot (Figure 1(a)). Then, the correlations between the expression levels of lncRNA XIST, miR-103a-3p, and HMGB1 mRNA were analyzed by Spearman's rank-order correlation test. The expression levels of lncRNA XIST and miR-103a-3p had a negative correlation (*P* < 0.01, *R*^2^ = 0.67), and the expression levels of HMGB1 mRNA and miR-103a-3p also had a negative correlation (*P* < 0.01, *R*^2^ = 0.69). A positive correlation between lncRNA XIST and HMGB1 mRNA expression was also found (Figure 1(b)) (*P* < 0.01, *R*^2^ = 0.70), which indicated that lncRNA XIST, miR-103a-3p, and HMGB1 might participate in the regulation of DVT. To further study the regulatory roles of lncRNA XIST, miR-103a-3p, and HMGB1 on HUVECs in DVT, IL-1*β* was added into the HUVEC culture medium to simulate the formation of DVT. Compared with the control group, the cell viability of HUVECs was inhibited significantly with the addition of 10 ng mL^−1^ IL-1*β* for 48 h, and cell viability was lower than 50% (*P* < 0.01) (Figure 1(c)). Therefore, the HUVECs treated with 10 ng mL^−1^ IL-1*β* for 48 h were used in the subsequent experiments. The results of qRT-PCR indicated that lncRNA XIST and HMGB1 mRNA were highly expressed (*P* < 0.01), and the expression level of miR-103a-3p was inhibited observably (*P* < 0.01) in HUVECs treated by IL-1*β* (Figure 1(d)). Then, lncRNA XIST was knocked down to detect its effect on HUVECs under IL-1*β* treatment. There is a significant enhancement of cell viability of HUVECs under si-XIST transfection (*P* < 0.01) compared with transfection of si-NC (Figure 1(e)). More importantly, si-XIST could distinctively improve the cell viability after IL-1*β* treatment (*P* < 0.01) (Figure 1(e)). In addition, the apoptosis of HUVECs was reduced significantly by si-XIST transfection compared with si-NC transfection after IL-1*β* treatment (*P* < 0.01) (Figure 1(f)). In addition, the expression levels of apoptotic proteins were determined to further evaluate the effect of lncRNA XIST on cell apoptosis. In detail, the expressions levels of proapoptotic Bax protein and cleaved caspase 3 were downregulated, and the expression of antiapoptotic protein Bcl-2 was upregulated in the si-XIST+IL-1*β* group in comparison with the si-NC + IL-1*β* group (*P* < 0.01) (Figure 1(g)). Finally, the level of TF was detected by commercial ELISA kit, and it was found that si-XIST reduced the TF level under IL-1*β* treatment (*P* < 0.01) (Figure 1(h)). These results indicated that the knockdown of lncRNA XIST attenuated the IL-1*β*-triggered apoptosis of HUVECs and the change of TF level.

### 3.2. lncRNA XIST Sponged miR-103a-3p

**Figure 2:**
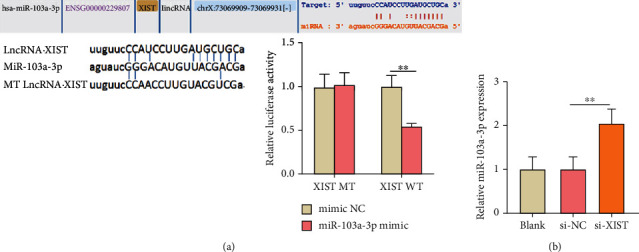
lncRNA XIST sponged miR-103a-3p in HUVECs. (a) Bioinformatic analysis using ENCORI database showed the interaction between lncRNA XIST with miR-103a-3p, and the binding relationship of lncRNA XIST and miR-103a-3p was verified by luciferase reporter gene assays. MT: mutant type; WT: wild type. (b) The expression of miR-103a-3p in transfected HUVECs was detected by qRT-PCR assays. ^∗^*P* < 0.05 and ^∗∗^*P* < 0.01.

The target relationship between lncRNA XIST and miR-103a-3p was forecasted by the ENCORI database (Figure 2(a)). In addition, the direct binding ability of lncRNA XIST on miR-103a-3p was verified by dual luciferase reporter assay. The luciferase activity in the XIST WT + miR-103a-3p mimic group was significantly decreased (*P* < 0.01). Meanwhile, the luciferase activity was almost unchanged in the other three groups, which revealed that lncRNA XIST could target miR-103a-3p (Figure 2(a)). Then, the relative expression level of miR-103a-3p was determined using qRT-PCR. It was obvious that lncRNA XIST knockdown could promote the expression level of miR-103a-3p significantly (*P* < 0.01) (Figure 2(b)).

### 3.3. miR-103a-3p Sponged HMGB1 and lncRNA XIST Could Increase the Expression of HMGB1 by Inhibiting miR-103a-3p

**Figure 3:**
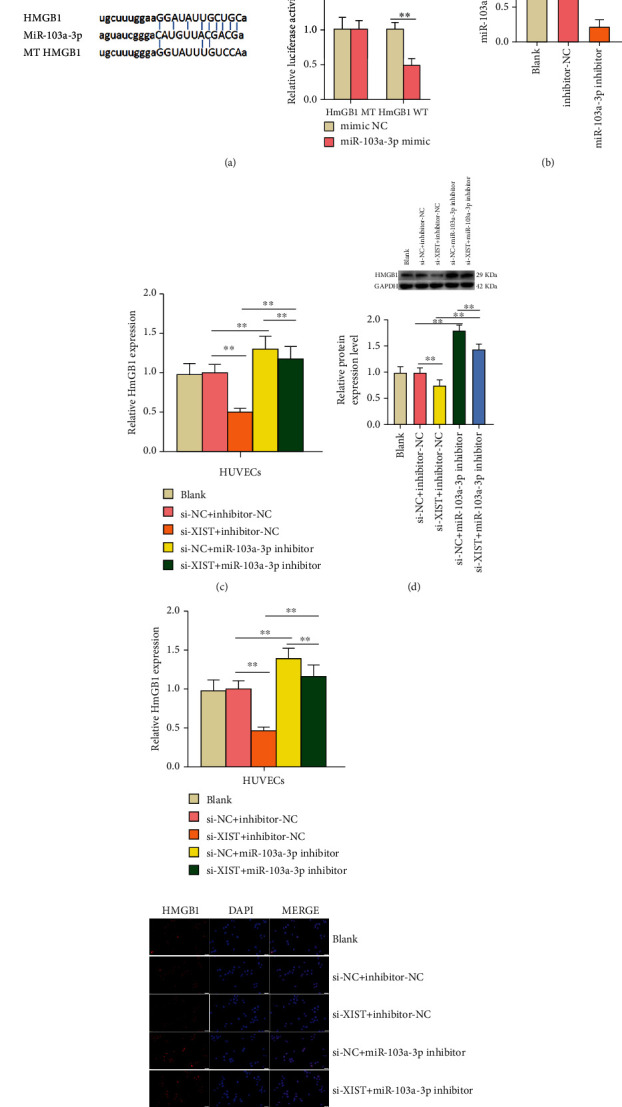
The expression of HMGB1 sponged by miR-103a-3p was improved by adding lncRNA XIST to sponge miR-103a-3p. (a) Bioinformatic analysis using ENCORI database shows the interaction of miR-103a-3p with HMGB1, and the binding relationship of miR-103a-3p and HMGB1 was proved by luciferase reporter gene assays. (b) The expression of miR-103a-3p in transfected HUVECs was detected by qRT-PCR. (c) The expression of HMGB1 in cotransfected cells was analyzed by qRT-PCR. (d) The expression level of HMGB1 protein in cotransfected cells was analyzed by western blot. (e) The expression level of HMGB1 protein in cotransfected cells was detected by immunofluorescence analysis (scale bar: 20 *μ*m). The nontrasfected HUVECs served as the blank group. ^∗^*P* < 0.05 and ^∗∗^*P* < 0.01.

The target relationship between HMGB1 and miR-103a-3p was predicted by the ENCORI database (Figure 3(a)). It was further verified with the dual luciferase reporter assay. The luciferase activity in the miR-103a-3p mimic+HMGB1 WT was significantly lower in contrast to the other three groups, which showed that miR-103a-3p could bind to HMGB1 (Figure 3(a)). To further explore the influence of miR-103a-3p on HMGB1 expression, the transfection efficiency of the miR-103a-3p inhibitor was detected. The results reported that the expression of miR-103a-3p was reduced significantly by adding miR-103a-3p inhibitor (*P* < 0.01) (Figure 3(b)). As shown in Figure 3(c), si-XIST inhibited the HMGB1 mRNA expression and miR-103a-3p inhibitor rescued the inhibitory effect of si-XIST on HMGB1 expression (*P* < 0.01). The result of western blot was consistent with the results of qRT-PCR (Figure 3(d)) (*P* < 0.01). Moreover, the results of the immunofluorescent assay further verified the effect of lncRNA XIST and miR-103a-3p on HMGB1 expression. Si-XIST inhibited the expression of HMGB1, and the addition of miR-103a-3p inhibitor can retard the inhibiting effects of si-XIST on HMGB1 expression (*P* < 0.01) (Figure 3(e)).

### 3.4. Knockdown of lncRNA XIST Alleviated the IL-1*β*-Induced Apoptosis and Change of TF Level by Regulating the ROS/NF-*κ*B Signal Pathway via miR-103a-3p/HMGB1 Axis

**Figure 4:**
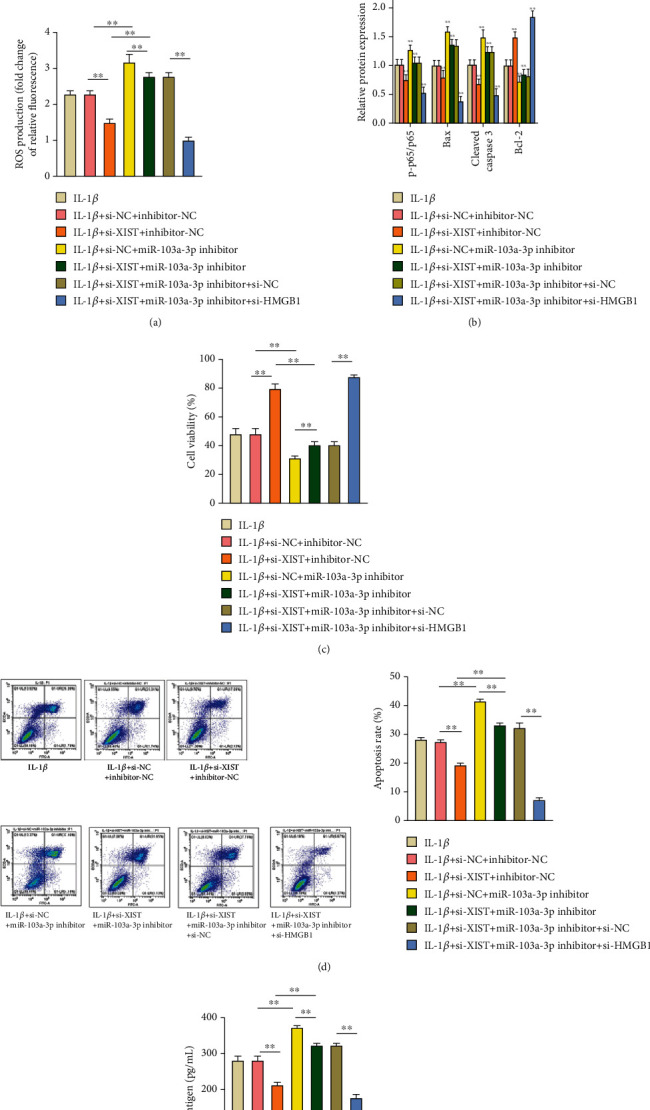
ROS-induced apoptosis and change of TF level based on adding IL-1*β* were regulated via lncRNA XIST/miR-103a-3p/HMGB1. (a) The ROS production in cotransfected cells was analyzed by DCFH-DA staining. (b) The expression levels of proteins related to the NF-*κ*B pathway and apoptosis in cotransfected cells were analyzed by western blot. (c) The viability of cotransfected cells was determined by MTT assay. (d) The apoptosis of cotransfected cells was tested by Annexin V-FITC/PI double staining kit. (e) The concentration of TF antigen in cotransfected cells was determined by the ELISA kit. ^∗^*P* < 0.05 and ^∗∗^*P* < 0.01.

To study the regulatory roles of the lncRNA XIST/miR-103a-3p/HMGB1 axis on apoptosis and TF level, the change of ROS level was exhibited in Figure 4(a). Compared with the IL-1*β* + si-NC + inhibitor-NC group, the ROS production was reduced significantly in the IL-1*β* + si-XIST+inhibitor-NC group and upregulated observably in the IL-1*β* + si-NC + miR-103a-3p inhibitor group (*P* < 0.01). The miR-103a-3p inhibitor could weaken the inhibitory effect of si-XIST on ROS production (*P* < 0.01). However, si-HMGB1 could reverse the influence of si-XIST and miR-103a-3p inhibitor on ROS production under IL-1*β* treatment (*P* < 0.01).

Then, to further explore the influence of ROS on apoptosis of HUVECs, the expression levels of the NF-*κ*B pathway-related proteins which were regulated by the ROS level, and the expression levels of apoptotic proteins were detected by western blot and shown in Figure 4(b). Si-XIST could inhibit the NF-*κ*B pathway, inhibits Bax and cleaved caspase 3 expressions, and promotes the expression levels of Bcl-2 under IL-1*β* treatment. However, the miR-103a-3p inhibitor could activate the NF-*κ*B pathway, inhibits Bcl-2 expression, and promotes the expression levels of Bax and cleaved caspase 3 under IL-1*β* treatment. miR-103a-3p inhibitor could rescue the inhibitory roles of si-XIST. Meanwhile, si-HMGB1 could reverse the combined effects of si-XIST and miR-103a-3p inhibitor (*P* < 0.01) under IL-1*β* treatment. Finally, the cell viability, apoptosis, and TF level indicating blood coagulation were evaluated (*P* < 0.01). miR-103a-3p inhibitor could promote apoptosis and inhibit cell viability, which can significantly reverse the influence of si-XIST (*P* < 0.01). Si-HMGB1 had a stronger effect on the inhibition of apoptosis and promoting cell viability via counteracting the effects of si-XIST plus miR-103a-3p inhibitor (*P* < 0.01) under IL-1*β* treatment (Figures 4(c) and 4(d)). Last but not least, for TF production, the miR-103a-3p inhibitor could reverse the inhibition of TF production caused by si-XIST under IL-1*β* incubation. Meanwhile, the promotion of TF production by adding miR-103a-3p inhibitor could be reversed by adding si-HMGB1 under IL-1*β* treatment (*P* < 0.01) (Figure 4(e)). These results above exhibited that knockdown of lncRNA XIST ameliorated IL-1*β*-triggered apoptosis and change of TF level in HUVECs by regulating the ROS/NF-*κ*B signaling pathway via miR-103a-3p/HMGB1 axis.

## 4. Discussion

DVT has been a common and serious CVDs in China. DVT patients suffered large torments for a long time due to the lack of practical therapies [1–3]. Therefore, the exploration of a novel and practical treatment was important. Some researchers found that the lncRNAs were great therapeutic targets based on their regulation in some diseases related to DVT [35, 36]. lncRNA MALAT1 was highly expressed in endothelial cells in diabetes [37]. Meanwhile, lncRNA MALAT1 was overexpressed in DVT tissues, which could regulate the proliferation and migration of EPC (the source of HUVECs) in DVT [38]. The high expression of lncRNA Sirt1-AS in HUVECs can improve the silent information regulator 1 (sirt1) to reduce the development of DVT associated with age [36]. Our research exhibited that the lncRNA XIST was expressed in abundance in the plasma of DVT patients compared with the plasma of normal people. There were similar reports in other cardiovascular diseases related to DVT [9–11]. In addition, we found that lncRNA XIST knockdown could increase cell viability and reduce apoptosis and ameliorate the change of TF level in HUVECs induced by IL-1*β*. The viability of HUVECs was improved upon XIST knockdown under oxidative low-density lipoprotein treatment in atherosclerosis [11]. The apoptosis of HUVECs was inhibited by lncRNA XIST knockdown via miR-30c-5p/PTEN axis in atherosclerosis [39]. The TF level associated with dilated cardiomyopathy was improved through lncRNA XIST/miR-195-5p/NOVA1 axis [40], and the high level of TF facilitated thrombi formation in DVT patients [41].

It was reported that the low miR-103a-3p level inhibited the migration and angiogenesis of EPC in DVT [12]. The high level of miR-103a-3p blocked the release of inflammatory factors and thrombus in acute LEDVT [14]. We found that miR-103a-3p had lower expression in DVT patients in comparison with the healthy volunteers, and the apoptosis and the level of TF in HUVECs were promoted by adding miR-103a-3p inhibitor under IL-1*β* treatment. Meanwhile, miR-103a-3p inhibitor reduced the cell viability under IL-1*β* treatment. The low expression of miR-103a-3p made a different contribution to different diseases. miR-103a-3p inhibitor increased apoptosis and autophagy to cause the injury of cardiomyocytes [42]. miR-103a-3p had a low expression level in endothelial cells in hypercholesterolemia [43]. In our study, the binding relationship of lncRNA XIST and miR-103a-3p was predicted and verified.lncRNA XIST sponged miR-103a-3p to regulate cell viability, apoptosis, and TF level in HUVECs in DVT.

It was found that HMGB1 was overexpressed in DVT tissues [15]. Our study evidenced that the level of HMGB1 was upregulated in DVT, and si-HMGB1 promoted the cell viability, inhibited the apoptosis, and ameliorated the change of TF level in HUVECs to block the development of DVT. Further, si-HMGB1 inhibited the ROS/NF-*κ*B signaling pathway and reduced ROS production to attenuate the function damage of HUVECs. There were similar reports on different diseases. The ROS production was inhibited upon HMGB1 silencing followed by erastin treatment in leukemia [44]. It was reported that the ROS/NF-*κ*B signaling pathway was inhibited which could attenuate the autophagy in the vascular smooth muscle cells in atherosclerosis [45]. Meanwhile, the inhibition of ROS/NF-*κ*B can reduce the apoptosis of HUVECs [26]. ROS could also induce the change of TF level in HUVECs [1, 25]. In our study, we found that the apoptosis and change of TF level in HUVECs were related to the regulation of the ROS/NF-*κ*B signaling pathway via the lncRNA XIST/miR-103a-3p/HMGB1 axis.

## 5. Conclusion

In this research, it was documented that lncRNA XIST knockdown could promote cell viability, inhibit apoptosis, and ameliorate the change of TF level in HUVECs induced by IL-1*β*. The ROS level was reduced, and ROS/NF-*κ*B signaling pathway was inhibited by lncRNA XIST knockdown via miR-103a-3p/HMGB1 axis, which could attenuate IL-1*β*-induced dysfunction of HUVECs to reduce the risk of DVT. Our study provided more information about the role of lncRNA XIST in HUVECs and established a fundamental basis for the development of target therapies for DVT in the future.

## Figures and Tables

**Table 1:** RNA oligonucleotides used for cell transfection.

RNA oligonucleotides	RNA oligonucleotides sequences
Si-NC	5′- UCU AGA AGU GCU UGC AGC A TT -3′
	3′- TT AGA UCU UCA CGA ACG UCG U -5′
Si-XIST	5′- GGA CCA GUU CAG UAC CAU A TT -3′
	3′- TT CCU GGU CAA GUC AUG GUA U -5′
Inhibitor-NC	5′ CUG CUA GAC GAU GCU GCU GCA-3′
	3′- GAC GAU CUG CUA CGA CGA CGU -5′
miR-130-3p inhibitor	5′- GAC CAG AGG UGU GCA GCU UUC -3′
	3′- CUG GUC UCC ACA CGU CGA AAG -5′
Si-NC	5′- UGU AGU UCA UCG UCU AGC A TT -3′
	3′- TT ACA UCA AGU AGC AGA UCG U -5′
Si-HMGB1	5′- GGA CCA CUA CAG AUG CAU A TT -3′
	3′- TT CCU GGU GAU GUC UAC GUA U -5′

**Table 2:** Primers used for qRT-PCR.

Genes	Primer sequences
XIST	Forward: 5′-CTACATTCCTGAGCCGTTATCT-3′
	Reverse: 5′-GGATGTAATCGACGTCTCTCAT-3′
miR-130-3p	Forward: 5′-TCCGCTGTAGAGATAGGC-3′
	Reverse: 5′-ATGGAGACTGAGGATCACTG-3′
HMGB1	Forward: 5′-TGCGACAAGATCGTGCATCC-3′
	Reverse: 5′-GCAGTAGAGCTTCTACTCGTAG-3′
U6	Forward: 5′-CTCGCTTCGACAGCACAGATACT-3′
	Reverse: 5′-ACGCTTCACGAACTTGCGTGTC-3′
GAPDH	Forward: 5′-CGCTCTCTGCTCCTACTGTTC-3′
	Reverse: 5′- ATCtGTTGACTCCGACATTCAC-3′

**Table 3:** Baseline characteristics of DVT patients and healthy controls.

Characteristics	Healthy controls (*n* = 20)	DVT patients (*n* = 20)
Age, years (mean ± SD)	49.24 ± 13.75	48.75 ± 14.24
Gender, females/males	9/11	9/11
BMI, kg/m^2^ (mean ± SD)	24.04 ± 4.47	23.96 ± 4.56
Hypertension	0	0
Diabetes mellitus	0	0
Other chronic diseases	0	0

## Data Availability

The experiment data used to support the findings of this study are available from the corresponding author upon reasonable request.

## Abbreviations

XIST: X inactive-specific transcript
DVT: Deep vein thrombosis
CVDs: Cardiovascular diseases
HUVECs: Human umbilical vein endothelial cells
lncRNAs: Long noncoding RNAs
miRNA: microRNA
mRNA: Messenger RNA
UTR: Untranslated regions
ENCORI: Encyclopedia of RNA interactomes
FOXO1: Forkhead box protein 1
TLR4: Toll-like receptor 4
EPCs: Endothelial progenitor cells
PTEN: Phosphatase and tensin homolog
LEDVT: Lower-extremity deep venous thrombosis
CXCL12: Chemokine 12
HMGB1: High-mobility group box 1
ROS: Reactive oxygen species
IL-1*β*: Interleukin-1beta
TF: Tissue factor
NF-*κ*B: Nuclear transcription factor-kappa B
HRP: Horseradish peroxidase.
